# Gene regulation in t(6;9) DEK::NUP214 Acute Myeloid Leukemia resembles that of FLT3-ITD/NPM1 Acute Myeloid Leukemia but with an altered HOX/MEIS axis

**DOI:** 10.1038/s41375-023-02118-1

**Published:** 2024-01-04

**Authors:** Sandeep Potluri, Sophie G. Kellaway, Daniel J. L. Coleman, Peter Keane, Maria Rosaria Imperato, Salam A. Assi, Peter N. Cockerill, Constanze Bonifer

**Affiliations:** 1 Institute of Cancer and Genomic Sciences, University of Birmingham, Birmingham, UK; 2 Present Address: Blood Cancer and Stem Cells, Centre for Cancer Sciences, School of Medicine, University of Nottingham, Nottingham, UK; 3 Present Address: School of Biosciences, University of Birmingham, Birmingham, UK

**Keywords:** Acute myeloid leukaemia, Cancer genomics, Oncogenes

## To the Editor:

Acute Myeloid Leukemia (AML) represents a heterogeneous group of hematological malignancies. The t(6;9)(p23;q34) translocation, generating the DEK::NUP214 fusion protein is found in 1% of AML. It causes a highly aggressive disease with poor prognosis in patients with a median age of just 23 [1]. t(6;9) AML often harbours a FLT3 internal tandem duplication (ITD) mutation as well which contributes to adverse outcomes [1]. Differing mechanisms underlying disrupted differentiation and proliferation in AML challenge treatment improvement, as each AML sub-type forms its own gene regulatory network (GRN) dependent on the driver mutation, which is distinct from healthy cells [2]. GRNs highlight which transcription factors (TFs) regulate which genes at which level, and inform on specific vulnerabilities [2]. Despite harbouring different driver oncogenes, gene expression patterns of t(6;9) AML resemble those of NPM1-mutated and NUP98::NSD1 AML [3, 4]. However, it remains unclear how DEK::NUP214 de-regulates gene expression in AML as the GRN has not been studied [5, 6].

In healthy hematopoietic cells, DEK is a DNA and RNA binding protein with different functions including modulating chromatin accessibility and histone acetylation [6]. NUP214 is a part of the nuclear pore complex, with roles in multiple pathways such as cell cycle progression and nucleocytoplasmic transport [5]. DEK::NUP214 is thought to disrupt various nuclear processes leading to the dysregulation of myeloid differentiation [6]. This includes deregulation of *HOX* gene clusters, which encode a family of TFs with crucial roles in normal hematopoietic development and which are tightly regulated display spatial-temporal expression patterns and are required for normal haematopoietic differentiation [7]. Aberrant activation of *HOX* genes has been associated with leukemogenesis in multiple AML sub-types, including t(6;9) [3, 4, 7, 8]. Here, we utilise genome-wide chromatin accessibility to elucidate how the normal haematopoietic progenitor cell GRN is disrupted by DEK::NUP214. We find that the DEK::NUP214 AML GRN is related to that of mutant NPM1 AML, but also displays an elevated leukemic stem cell signature, suggesting overlapping and unique therapeutic vulnerabilities.

**Fig. 1:**
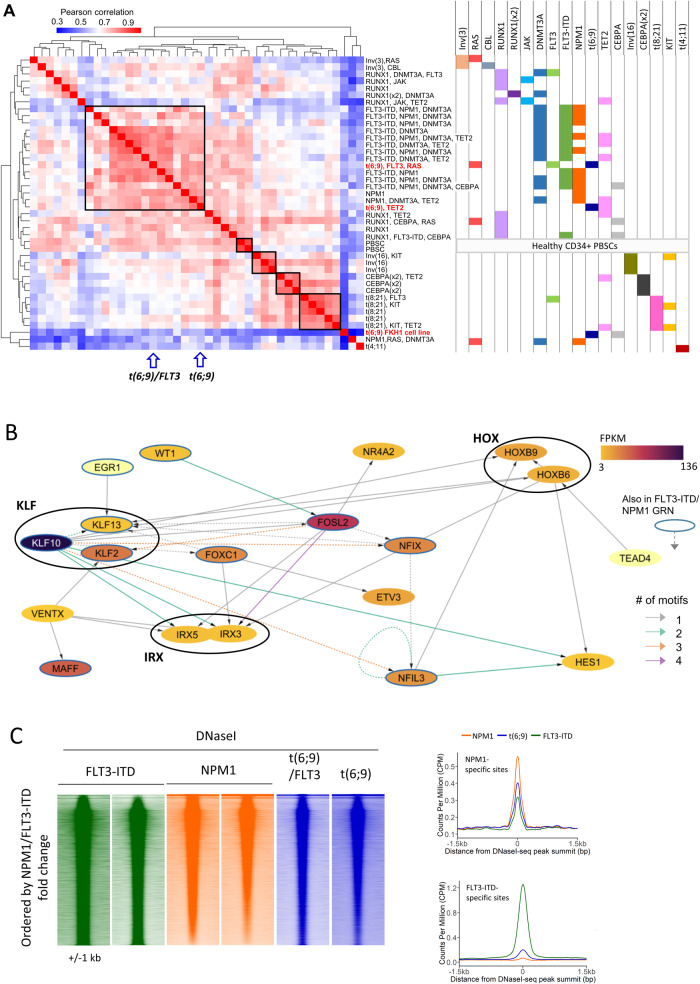
t(6;9) AML shares chromatin accessibility and gene expression with FLT3-ITD and NPM1-mutated AML. **A** Heatmap with hierarchical clustering showing the Pearson correlation of the tag counts at distal DNaseI peaks of t(6;9) patient blasts together with blasts from other mutational backgrounds and a t(6;9) cell line (FKH1). Mutations present in each sample are indicated to the right with common drivers in clusters shown on the heatmap. **B** t(6;9) AML-specific GRN, based on merged t(6;9)-specific DNase1 and RNA-seq data vs PBSCs. The colour of the node indicates the gene expression in FPKM, the edge colour shows how many sites with the source node motif regulate the target gene with the arrow providing directionality between source and target. Nodes outlined in blue and dashed edges are also present in the FLT3-ITD/NPM1 GRN. **C** Tag counts of the DNaseI distal peaks were ranked by the fold change between the average of two NPM1-only patients and two FLT3-ITD-only patients. Density plots show the tag counts across a 2 kb window, with the t(6;9) patients plotted on the same axis. Average profiles (right) show the average signal of DNaseI in the t(6;9), NPM1 and FLT3-ITD patients across all the 4-fold FLT3-ITD or NPM1 specific sites.

To address the question of how gene expression is de-regulated in t(6;9) AML, we assessed chromatin accessibility using DNaseI-seq and gene expression by RNA-seq in CD34-purified AML blasts from two t(6;9)-positive patients and a t(6;9) cell line, FKH1. One patient also carried a FLT3 tyrosine kinase domain mutation. The pattern of DNaseI hypersensitive sites (DHSs) was similar between the two patients (Fig. 1A, Supplementary Fig. 1A). Unsupervised clustering of this data together with previously generated DNaseI-seq data from patients with other genotypes [2, 9], showed that t(6;9) AML is part of the larger FLT3-ITD, NPM1 and FLT3-ITD/NPM1 AML cluster (Fig. 1A). Chromatin accessibility data for patients with UBTF-TD, KMT2A-PTD and NUP98-rearranged AML known to deregulate *HOX* genes were not available. FKH1 proved to be an unsuitable model for t(6;9) AML as its DHS pattern showed little correlation with any primary AML blasts (Fig. 1A), therefore downstream analysis used only patient cell DNaseI data. AML-specific gene expression, determined by RNA-seq on the t(6;9) primary AML samples compared to healthy peripheral blood stem cells (PBSCs), varied between the patients but showed a significant overlap of deregulated genes (p = 7.7 × 10^−66^ upregulated genes, p = 1.3 × 10^−99^ downregulated genes; Supplementary Fig. 1B). Inspection of known FLT3-ITD and NPM1 de-regulated genes showed similar expression in t(6;9) AML (Supplementary Fig. 1C). To construct a t(6;9)-specific GRN, we used merged DNaseI and RNA-seq data from both patients filtered against PBSCs, with pan-AML promoter-capture Hi-C to confidently assign enhancers to their cognate genes, as previously described [10]. This analysis showed that t(6;9) AML shares key regulatory TF nodes with FLT3-ITD and NPM1 AML such as *NFIL3*, *FOXC1, NFIX, WT1, EGR1* and AP-1 (*FOSL2*), which are essential for FLT3-ITD/NPM1 AML maintenance [2, 10] and which were also de-regulated in t(6;9) AML (Fig. 1B, Supplementary Fig. 1C).

To evaluate the regulatory phenotype of t(6;9) as compared to FLT3-ITD or NPM1 AML, we ranked the DNaseI data by the fold-change of the DHS tag count between NPM1 and FLT3-ITD patients, and plotted the t(6;9) DNaseI signal alongside (Fig. 1C, left). These analysis showed that the DHS pattern in t(6;9) AML closely resembles that of NPM1-mutated AML. Clustering just t(6;9), FLT3-ITD and NPM1 DHS data (Supplementary Fig. 1D) and plotting the average signal across NPM1 or FLT3-ITD specific DHSs (Fig. 1C, right), showed that t(6;9) patient chromatin was accessible at the NPM1- but not the FLT3-ITD-AML specific sites. To identify TFs mediating this pattern, we employed digital footprinting followed by de novo motif discovery. TF binding motifs enriched in both sets of t(6;9) specific footprints as compared to those of healthy PBSCs (Supplementary Fig. 1E) included occupied EGR, NF1 and AP-1 motifs, confirming that these core nodes of the GRN are involved in regulating t(6;9)-specific AML gene expression. Footprints were also enriched with RUNX, ETS and C/EBP motifs which are bound by these global haematopoietic regulators. Together these data show that the GRN driven by DEK::NUP214 is similar to FLT3-ITD and closest to that of NPM1-driven AML, but does not completely overlap.

**Fig. 2:**
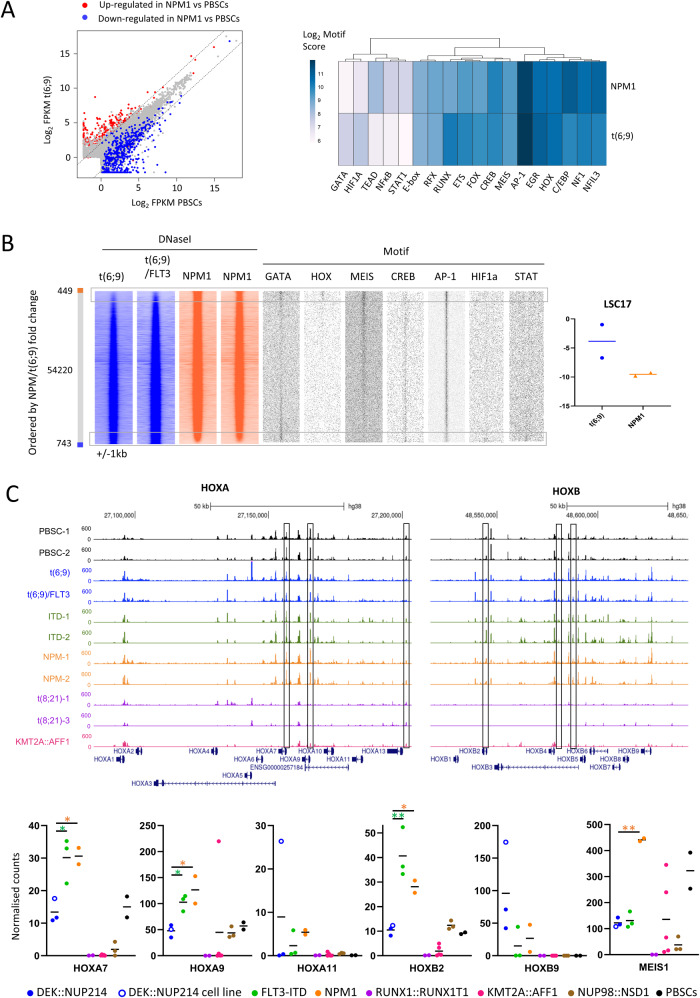
t(6;9) AML differs from NPM1-mutated AML in the HOX/MEIS axis. **A**. Scatterplot of gene expression as log_2_ FPKM in t(6;9) vs PBSCs. Genes shown in red are at least 4-fold upregulated in blue at least 4-fold downregulated in NPM1 AML compared to PBSCs. Dashed lines de-lineate 4-fold up and downregulated in t(6;9) vs PBSCs. Heatmap with hierarchical clustering showing the log_2_ motif score for footprinted motifs identified in t(6;9) and NPM1 DNase1 datasets and not found in PBSCs. **B** Tag counts of the DNaseI distal peaks were ranked by the fold change between the average of two NPM1-only patients and two t(6;9)-only patients. Density plots show the tag counts across a 2 kb window, with enriched motifs plotted on the same axis. LSC17 scores were calculated from the normalised FPKM values of the RNA-seq for these patients, the horizontal bar indicates the mean of the two patients which are shown by individual points. **C** UCSC Genome Browser screenshot of the *HOXA* and *HOXB* loci showing normalised DNaseI read coverage, boxes indicate sites which display less accessible chromatin in both t(6;9) patients as compared to all FLT3-ITD and NPM1 patients, average 1.94-fold less accessible across all peaks in each patient compared to each FLT3-ITD/NPM1 patient (range 1.17–5.49-fold), top. Normalised counts of key *HOX* genes and *MEIS1* shown for cells from t(6;9) patients and a t(6;9) cell line, healthy PBSCs and patient cells from other AML backgrounds. Horizontal line indicates the mean, individual patients/cell line are shown by circles. *indicates *p* < 0.05, **p < 0.01 with green asterisks showing the significance between t(6;9) and FLT3-ITD only expression and orange asterisks showing significance between t(6;9) and NPM1 only expression by Student’s T-test.

NPM1-mutated AML is associated with a more favourable prognosis than t(6;9) AML. We therefore evaluated the differences in gene regulation underpinning this phenotype. Whilst t(6;9) and NPM1 AML gene expression patterns (Fig. 2A, left) and the enrichment of footprinted TF motifs such as for AP-1 and HOX (Fig. 2A, right) were overall similar, differences were evident. To investigate these, we this time ranked the DHS tag counts by the fold change between NPM1 and t(6;9) (Fig. 2B) demonstrating that NPM1-specific DHSs were enriched with HOX and MEIS motifs. In contrast, HIF1A and STAT motifs were enriched in t(6;9) specific sites (Fig. 2B) which suggests increased signalling activity in t(6;9) patients and could drive leukemic stem cell (LSC) growth [11]. This notion was supported by a higher LSC17 score, a gene signature indicating the stemness and associated prognosis of an AML (Fig. 2B, right) in t(6;9) patients. Although NPM1 patients typically lack CD34 cell surface marker expression, the same trend was seen when excluding *CD34* from the calculation (Supplementary Fig. 2A). The poorer prognosis of t(6;9) AML patients may therefore be due to higher LSC numbers or growth but further investigation would be needed to test this hypothesis.

The HOX binding motifs in the NPM1-specific DHSs could be bound by any of several HOX factors. The *HOXA* and *HOXB* clusters, together with *MEIS1* are upregulated in NPM1 AML which contributes to the differentiation block [8]. *HOX* genes have also been reported as upregulated in t(6;9) AML as compared to other subtypes such as t(8;21) [4]. MLL-Menin modulates upregulation of *HOX* genes in NPM1-mutated and SET::NUP214 AML [5, 12], whilst other NUP fusion proteins cause upregulation of *HOX* genes [3] through co-operation of NUP proteins with *XPO1* (CRM1) [13] enriched at *HOX* promoters [14]. In line with these findings, the *HOX* loci of t(6;9) patients display accessible chromatin (Fig. 2C). However, accessibility at the *HOX* loci of t(6;9) patients differed when compared to that of patients carrying the FLT3-ITD without the NPM1 mutation (with additional mutations in *BCOR, DNMT3A, TET2, TP53* and Tri(13)), and patients carrying NPM1 mutation but not the FLT3-ITD. Patients with t(6;9) AML displayed less accessible chromatin at several *HOX* DHSs, including at the *HOXA7*, *HOXA9* and *HOXB2* promoters and at distal cis-regulatory elements (Fig. 2C). Moreover, mRNA expression of *HOXA7*, *HOXA9*, *HOXA11* (in both patient samples but not FKH1 cells) and *HOXB2* genes were significantly lower in t(6;9) AML compared to FLT3-ITD and NPM1 AML (Fig. 2C, Supplementary Table 1), although still upregulated and more accessible than in, for example, t(8;21) AML. Of the AMLs studied and with the exception of *HOXB9*, *HOX* gene expression patterns in t(6;9) AML most closely resembled NUP98::NSD1 [2, 9, 15].

*HOX* genes contribute to maintaining an immature phenotype of NPM1 AML [8], but compared to DHS patterns from healthy stage-specific datasets, NPM1, FLT3-ITD and t(6;9) AML showed similar maturity (Supplementary Fig. 2B). Therefore, specific *HOX* expression patterns are due to the driver mutation, rather than the cell stage of the differentiation block. Genes with HOX and/or MEIS sites at associated DHSs included *CD34*, which displays higher gene and cell surface expression in t(6;9) AML compared to NPM1 AML (Supplementary Fig. 2C, D). However, the majority of differentially expressed genes with HOX/MEIS motifs, including lymphoid genes, *MEIS1* and AP-1 family member genes, were expressed at least 2-fold higher in NPM1 patients (Supplementary Fig. 2C, Supplementary Table 1) suggesting reduced reliance on HOX factors in t(6;9) AML. Taken together, these data indicate differential regulation of the *HOX/MEIS* genes in t(6;9) AML as compared to NPM1 AML, with downstream HOX/MEIS binding sites also differentially regulated.

In conclusion, our study sheds light on the unique molecular characteristics of t(6;9) AML. It reveals that t(6;9) AML exhibits a GRN comparable to that of the NPM1 and FLT3-ITD AML subtypes but shows altered *HOX* expression and diminished downstream regulation by HOX-related pathways. However, t(6;9) AML exhibits a stronger LSC signature. These findings emphasize the need for tailored therapeutic approaches based on the broader gene regulatory program to target this highly aggressive AML sub-type. We have recently shown that targeting essential components in FLT3-ITD/NPM1 AML GRN, such as AP-1, FOXC1 or NFIX interferes with AML maintenance [2, 10]. We also have shown that LSC growth can be specifically inhibited [11]. Our data here indicate that similar strategies could be considered for t(6;9) AML.

### Supplementary information

Supplementary Information

Supplementary Table 1

## Data Availability

RNA-seq and DNaseI-seq produced in this study have been deposited in the Gene Expression Omnibus (GEO) under accession code GSE240272.
