# Supplementary material for: Gene regulation in t(6;9) DEK::NUP214 Acute Myeloid Leukemia resembles that of FLT3-ITD/NPM1 Acute Myeloid Leukemia but with an altered HOX/MEIS axis
Source: Leukemia. 2024 Jan 4;38(2):403–7. doi: 10.1038/s41375-023-02118-1 (PMC10844093; doi:10.1038/s41375-023-02118-1)
Supplement: Supplementary file 1 — Supplementary Information [file 41375_2023_2118_MOESM1_ESM.docx]

**Methods**

**Patient samples**

Human peripheral blood (sample labelled t(6;9)) or bone marrow (sample labelled t(6;9)/FLT3) was obtained with the required ethical approval from the NHS National Research Ethics Committee, with informed consent from patients from the Centre for Clinical Haematology, Queen Elizabeth Hospital Birmingham, UK. Sample t(6;9)/FLT3 had mutations in CHK2 T367fs, ETV6 Q150X, FLT3 D835H, NRAS G12D and ZRS2 K106X alongside the translocation. Sample t(6;9) had mutations in TET2 L34F (VUS) and EZH2 D185H alongside the translocation. Mononuclear cells were purified on the day the sample was received by density centrifugation using Lymphoprep (Stemcell Technologies) and sorted for CD34 using MACS.

**Cell culture**

FKH1 cells were obtained from DSMZ and routinely tested for mycoplasma. Cells were cultured in RPMI-1640 with 20% foetal bovine serum, 2mM L-glutamine and 1% Penicillin/Streptomycin and routinely split to 0.5 x 10^6^ every 4 days.

**Flow cytometry**

Flow cytometry was performed on samples post-lymphoprep before CD34-sorting using a PE-conjugated antibody against CD34 (Miltenyi Biotec,130-120-515) and the PE-conjugated isotype control. Samples were run on a CyanADP (Beckman Coulter), collected using Summit v4.3 and analysed using FlowJo v10.

**DNaseI-seq**

DNaseI digestions were performed as previously described.^1^ Briefly, cells were permeabilised and digested with DNaseI (Worthington) for 3 minutes at 22°C, and the reactions were terminated by addition of SDS to 0.5%. DNA fragments between 250 and 350 bp were selected and DNaseI-seq libraries were prepared using the KAPA hyper prep kit and sequenced on an Illumina NextSeq 500.

**RNA-seq**

RNA was extracted using Trizol. Sequencing libraries were prepared using the Illumina TruSeq Stranded Total RNA kit with Ribo-Zero Human/Mouse/Rat and sequenced on an Illumina NextSeq 500.

**DNaseI-seq analysis**

Data for non-t(6;9) AML samples was downloaded from the Gene Expression Omnibus accession GSE108316^2^ and GSE130142^3^, and for healthy haematopoietic cells from GSE74912^4^.

Sequencing reads were processed with Trimmomatic v0.32^5^ to remove sequencing adaptors and low-quality sequences, then aligned to hg38 with Bowtie2 v2.2.3^6^ using the setting –very-sensitive-local. PCR duplicates were removed using the MarkDuplicates function in Picard 2.10.5. Peaks were called using MACS2 2.1.1^7^ using the options -q 0.0005 -B –trackline –nomodel –shift 100 –extsize 200. Distal peaks were defined as being at least 1500 bp from the nearest transcription start site as annotated by HOMER v4.11^8^.

Clustering was carried out on a peak union annotated with tag counts using HOMER^8^ as described previously^2^. Tag counts were normalised using DESeq2, followed by upper quartile normalisation. A pseudocount of 1 was added prior to log_2_ transformation, Pearson correlation was calculated and then hierarchically clustered.

To carry out differential chromatin accessibility analysis, a peak union was generated using the bedtools v2.29.2^9^ merge function. The average tag-density in a 400-bp window centred on the peak union summits was calculated for each sample using the annotatePeaks.pl function in HOMER v4.11^8^ using the bedGraph files generated by MACS2^7^. These were then normalised as CPM and further log2-transformed as log2(CPM + 0.1). Peaks were considered as differentially accessible if there was at least a 2 or 4-fold difference between samples.

Density plots were generated using HOMER v4.11^8^ annotatePeaks.pl function using the bedGraph files generated by MACS2^7^, with the options -size 2000 -hist 10 -ghist, or using the motif position weight matrix files provided with HOMER and the additional option -m. The resulting files were then plotted using JavaTreeView 1.1.6.

Average profiles were generated by first producing bigwig files using the bamCoverage function in deepTools 3.5.0^10^ normalised as CPM, then calculating average peak heights with computeMatrix also in deepTools and plotted using R.

Footprinting was carried out on high read depth DNaseI using the Wellington method in pyDNase.^11^ Footprints which were specific to each t(6;9) sample individually as compared to a healthy PBSC sample were identified. De novo motif discovery was performed on these sites using HOMER. Average cut profiles were plotted using pyDNase.

To calculate a motif score, the number of motifs in footprints was first counted by extracting the motif positions using the annotatePeaks.pl function in HOMER with the options -size given -mbed. The probability weight matrices provided by the HOMER motif database were used in all analyses. Only motifs that were found in footprints were retained. The enrichment score for each footprinted set of motifs in each AML subtype was then calculated as:

$$S_{ij}= \frac{n_{ij}}{{\sum_{i} n_{ij}}/M}$$

Where n_ij_ represents the number of motifs i in AML subtype j. The sum of n_ij_ represents the sum of all motifs found in that AML subtype and M corresponds to the total number of motifs found across all AML subtypes examined. The scores were then clustered and plotted as a heatmap as for correlations. Motifs were identified for use in this analysis by first performing de novo motif discovery in HOMER.

Rolling averages of motif density with and without footprints were calculated by finding all of the given motif in a union of all DNaseI peaks across t(6;9) and NPM1 AML samples using annotatePeaks.pl function in HOMER with the options -size 200 -mbed. These motif coordinates were then assigned to their gene utilising pan-AML Hi-C data from GSE108316^2^ or nearest gene if Hi-C linked was not available. The average number of motifs per 1000 genes was then calculated based on gene expression ranked by fold change between t(6;9) and NPM1 in a moving window and plotted using R.

To generate normalised tracks for UCSC genome browser, the average peak height was calculated across a union of all peaks in all samples for each sample and all samples from average profiles as above. A normalisation factor was then calculated as sample average/overall average. Bigwig files were then re-generated using bamCoverage in deepTools with normalisation according to the normalisation factor.

**RNA-seq analysis**

Data for FLT3-ITD, NPM1, t(8;21) and healthy PBSC samples were downloaded from the Gene Expression Omnibus accession GSE108316^2^, for KMT2A::AFF1 from GSE132396^3^ and raw counts were provided by the study authors for NUP98::NSD1 patient cells following 7 days of culture with vehicle control^12^.

Raw paired-end sequencing reads were processed with Trimmomatic v0.32^5^ to remove sequencing adaptors and low-quality sequences, then aligned to hg38 with Hisat2 2.1.0^13^ with default parameters. Gene expression values were calculated as fragments per kilobase of transcript per million mapped reads (FPKM) using Stringtie 1.3.3^14^.

To compare between different AML subtypes, counts were obtained using FeatureCounts^15^ within the Subread package v2.0.1 using the options -p -B -s2 and gene models from Ensembl as the reference transcriptome. Only genes with at least 10 counts in at least one sample were retained for further analysis. Counts were normalized using the edgeR package in R v4.1.0, and differential gene expression analysis was then carried out using limma-voom^16^. Unpaired two-side T-tests were used to calculate p-values.

Significance of overlap between t(6;9) specific genes vs PBSCs within the two patients was calculated using hypergeometric testing.

LSC17 score was calculated as previously described^17^, using normalised FPKM values, but also calculated by omitting CD34 from the equation to assess if this was solely contributing to differences observed between CD34- NPM1 patients and CD34+ t(6;9) patients.

**Gene Regulatory Network construction**

The gene regulatory network was made using publicly available Python scripts as previously described.^18^ Briefly, 4-fold specific DNaseI hypersensitive sites (DHS) were selected based on the average normalised peak height across the two t(6;9) samples compared to two healthy PBSC samples as above. Genomic coordinates of transcription factor binding motifs were then retrieved in this peak set using annotatePeaks.pl function in HOMER^8^. These motif coordinates were then assigned to their gene utilising pan-AML Hi-C data from GSE108316^2^ or nearest gene if Hi-C linked was not available. Only transcription factors which were expressed at least 4-fold higher in t(6;9) compared to PBSCs were retained. A network was then constructed where the transcription factor genes are represented by nodes and the presence of a motif for transcription factor 1 in a DHS targeting transcription factor 2 represented by a directed edge. Members of the same transcription factor family with a shared motif were grouped. The network was visualised using Cytoscape 3.9.1.

**Supplementary Figures**


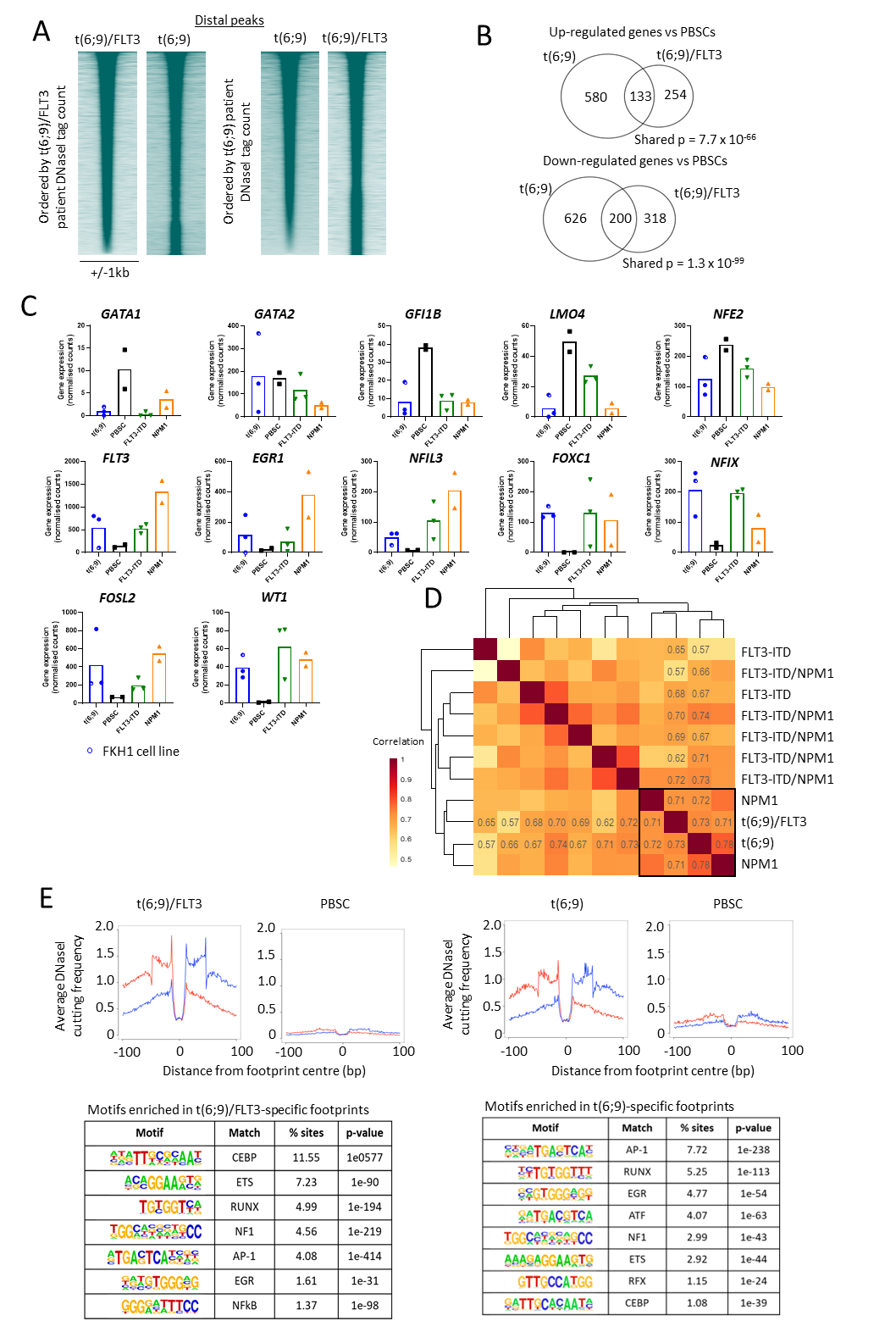


**Supplementary Figure 1: t(6;9) AML shares chromatin accessibility and gene expression with FLT3-ITD and NPM1-mutated AML.**

**A.** Density plots showing the distal DNaseI peaks for each sample ranked by the tag count and plotted across a 2kb window. In each case, the tag counts of the non-ranked sample are plotted alongside on the same axis. Left panels are ranked by the tag count of t(6;9)/FLT3 sample, right panels are ranked by the tag count of the t(6;9) sample. **B.** Venn diagrams showing the number of 3-fold upregulated genes in each t(6;9) sample as compared to healthy PBSCs, and the overlap between the two t(6;9) samples. The p-value of whether the overlap is higher than expected by chance as determined by hypergeometric testing is indicated. **C.** Normalised counts for known FLT3-ITD or NPM1 associated genes, the bar indicates the mean of the individual patients shown by the points, the unshaded point on the t(6;9) graph indicates the FKH1 cell line.  **D.** Heatmap with hierarchical clustering showing the Pearson correlation of the tag counts at distal DNaseI peaks in t(6;9), FLT3-ITD alone, NPM1 alone and FLT3-ITD/NPM1 patients. The correlation coefficient is shown in each box for the t(6;9) samples vs all others. **E.** Average profiles of DNaseI cut sites at t(6;9) specific footprints in t(6;9) and healthy PBSC samples. Motifs found in these footprints are shown below.


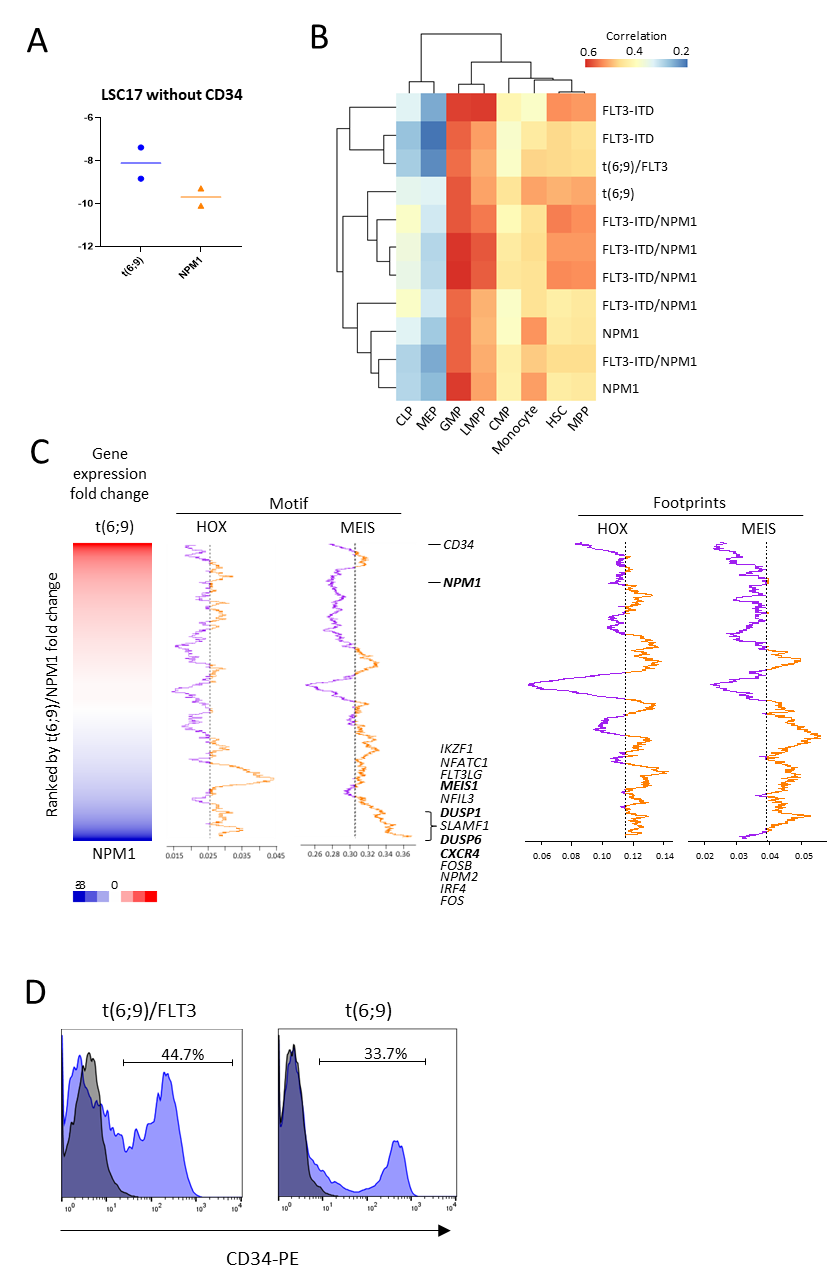


**Supplementary Figure 2: t(6;9) AML differs from NPM1-mutated AML in the HOX/MEIS axis.**

**A.** LSC17 scores were calculated from the normalised FPKM values of the RNA-seq for these patients excluding any contribution by CD34, the horizontal bar indicates the mean of the two patients which are shown by individual points. **B.** Heatmap with hierarchical clustering showing the Pearson correlation of the tag counts at distal DNaseI peaks in t(6;9), FLT3-ITD alone, NPM1 alone and FLT3-ITD/NPM1 patients, with peaks specific to each haematopoietic differentiation stage.^3^ **C.** Fold change of gene expression was ranked by the difference between the average of the t(6;9) patients and the average of the NPM1 alone patients. The rolling average of the presence or absence of HOX and MEIS motifs (left) or footprinted motifs (right) in distal sites associated with these genes is plotted alongside. The dotted line indicates the average number of motifs across the whole ranking. Genes of interest which are at least 2-fold up or downregulated and have either a HOX or MEIS motif are marked, and those with a footprinted motif are shown in bold. **D.** Flow cytometry showing CD34-PE (blue) overlaid with isotype control-PE (grey) for t(6;9) patients following lymphoprep, prior to cell sorting.

**References**

1. Bert AG, Johnson BV, Baxter EW, Cockerill PN. A modular enhancer is differentially regulated by GATA and NFAT elements that direct different tissue-specific patterns of nucleosome positioning and inducible chromatin remodeling. Mol Cell Biol. 2007 Apr;27(8):2870-85. doi: 10.1128/MCB.02323-06
2. Assi SA, Imperato MR, Coleman DJL, Pickin A, Potluri S, Ptasinska A, Chin PS, Blair H, Cauchy P, James SR, Zacarias-Cabeza J, Gilding LN, Beggs A, Clokie S, Loke JC, Jenkin P, Uddin A, Delwel R, Richards SJ, Raghavan M, Griffiths MJ, Heidenreich O, Cockerill PN, Bonifer C. Subtype-specific regulatory network rewiring in acute myeloid leukemia. Nat Genet. 2019 Jan;51(1):151-162. doi: 10.1038/s41588-018-0270-1
3. Tirtakusuma R, Szoltysek K, Milne P, Grinev VV, Ptasinska A, Chin PS, Meyer C, Nakjang S, Hehir-Kwa JY, Williamson D, Cauchy P, Keane P, Assi SA, Ashtiani M, Kellaway SG, Imperato MR, Vogiatzi F, Schweighart EK, Lin S, Wunderlich M, Stutterheim J, Komkov A, Zerkalenkova E, Evans P, McNeill H, Elder A, Martinez-Soria N, Fordham SE, Shi Y, Russell LJ, Pal D, Smith A, Kingsbury Z, Becq J, Eckert C, Haas OA, Carey P, Bailey S, Skinner R, Miakova N, Collin M, Bigley V, Haniffa M, Marschalek R, Harrison CJ, Cargo CA, Schewe D, Olshanskaya Y, Thirman MJ, Cockerill PN, Mulloy JC, Blair HJ, Vormoor J, Allan JM, Bonifer C, Heidenreich O, Bomken S. Epigenetic regulator genes direct lineage switching in MLL/AF4 leukemia. Blood. 2022 Oct 27;140(17):1875-1890
4. Corces MR, Buenrostro JD, Wu B, Greenside PG, Chan SM, Koenig JL, Snyder MP, Pritchard JK, Kundaje A, Greenleaf WJ, Majeti R, Chang HY. Lineage-specific and single-cell chromatin accessibility charts human hematopoiesis and leukemia evolution. Nat Genet. 2016 Oct;48(10):1193-203. doi: 10.1038/ng.3646
5. Bolger, A.M., Lohse, M. & Usadel, B. Trimmomatic: a flexible trimmer for Illumina sequence data. Bioinformatics. 2014. 30, 2114-2120
6. Langmead, B. & Salzberg, S.L. Fast gapped-read alignment with Bowtie 2. Nature Methods. 2012. 9, 357-359
7. Zhang Y, Liu T, Meyer CA, Eeckhoute J, Johnson DS, Bernstein BE, Nusbaum C, Myers RM, Brown M, Li W, Liu XS. Model-based Analysis of ChIP-Seq (MACS). Genome Biology. 2008 9, R137
8. Heinz S, Benner C, Spann N, Bertolino E, Lin YC, Laslo P, Cheng JX, Murre C, Singh H, Glass CK. Simple Combinations of Lineage-Determining Transcription Factors Prime cis-Regulatory Elements Required for Macrophage and B Cell Identities. Molecular Cell. 2010. 38, 576-589
9. Quinlan, A.R. & Hall, I.M. BEDTools: a flexible suite of utilities for comparing genomic features. Bioinformatics. 2010. 26, 841-842
10. Ramírez F, Ryan DP, Grüning B, Bhardwaj V, Kilpert F, Richter AS, Heyne S, Dündar F, Manke T. deepTools2: a next generation web server for deep-sequencing data analysis. Nucleic Acids Res. 2016. 44, W160-W165.
11. Piper J, Elze MC, Cauchy P, Cockerill PN, Bonifer C, Ott S. Wellington: a novel method for the accurate identification of digital genomic footprints from DNase-seq data. Nucleic Acids Res. 2013 Nov;41(21):e201. Erratum in: Nucleic Acids Res. 2014;42(17):11272.
12. Rasouli M, Blair H, Troester S, Szoltysek K, Cameron R, Ashtiani M, Krippner-Heidenreich A, Grebien F, McGeehan G, Zwaan CM, Heidenreich O. The MLL-Menin Interaction is a Therapeutic Vulnerability in NUP98-rearranged AML. Hemasphere. 2023 Jul 27;7(8):e935
13. Kim, D., Paggi, J.M., Park, C., Bennett, C. & Salzberg, S.L. Graph-based genome alignment and genotyping with HISAT2 and HISAT-genotype. Nature Biotechnology. 2019. 37, 907-915
14. Pertea M, Pertea GM, Antonescu CM, Chang TC, Mendell JT, Salzberg SL. StringTie enables improved reconstruction of a transcriptome from RNA-seq reads. Nature Biotechnology. 2015. 33, 290-295
15. Liao, Y., Smyth, G.K. & Shi, W. featureCounts: an efficient general purpose program for assigning sequence reads to genomic features. Bioinformatics. 2013.30, 923-930
16. Ritchie ME, Phipson B, Wu D, Hu Y, Law CW, Shi W, Smyth GK. limma powers differential expression analyses for RNA-sequencing and microarray studies. Nucleic Acids Res. 2015. 43, e47-e47
17. Ng SW, Mitchell A, Kennedy JA, Chen WC, McLeod J, Ibrahimova N, Arruda A, Popescu A, Gupta V, Schimmer AD, Schuh AC, Yee KW, Bullinger L, Herold T, Görlich D, Büchner T, Hiddemann W, Berdel WE, Wörmann B, Cheok M, Preudhomme C, Dombret H, Metzeler K, Buske C, Löwenberg B, Valk PJ, Zandstra PW, Minden MD, Dick JE, Wang JC. A 17-gene stemness score for rapid determination of risk in acute leukaemia. Nature. 2016 Dec 15;540(7633):433-437
18. Coleman DJL, Keane P, Luque-Martin R, Chin PS, Blair H, Ames L, Kellaway SG, Griffin J, Holmes E, Potluri S, Assi SA, Bushweller J, Heidenreich O, Cockerill PN, Bonifer C. Gene regulatory network analysis predicts cooperating transcription factor regulons required for FLT3-ITD+ AML growth. bioRxiv 2023.07.18.549495
